# Editorial Comment on Special Issue—COVID-19 after One Year of Clinical Experience: Unexpected Clinical Presentations or Complications and Post-COVID-19 Clinical Features

**DOI:** 10.3390/healthcare10091715

**Published:** 2022-09-07

**Authors:** Pierpaolo Di Micco, Anna Annunziata, Giuseppe Fiorentino

**Affiliations:** 1 UOC Medicina, PO A. Rizzoli, ASL Napoli 2 Nord, Lacco Ameno, 80076 Naples, Italy; 2 Department of Respiratory Pathophysiology and Rehabilitation Monaldi–A.O. Dei Colli, 80131 Naples, Italy

Beginning in 2020, the COVID-19 pandemic caused by SARS-CoV-2 remains ongoing. However, in 2022 this virus has a different diffusion in the population compared to at the beginning of pandemic. Together with personal safety measures that aimed to reduce environmental transmission (e.g., wearing a face mask, reduced numbers of people in public areas, frequent cleaning of hands, and climatic variation and circulation of virus), the COVID-19 vaccination campaign gradually reduced the number of patients infected with severe COVID-19 (i.e., lung failure with increased morbidity, mortality and hospitalization rate). However, mortality is frequently associated with the presence of specific risk factors [1,2].

Yet, regarding the presence of viral variants of concern (VOCs), associated with immunization against the spike protein, the vaccination campaign actually reduced not only the severity of the disease and the clinical approach to infected patients but also clinical signs and symptoms of infection. In this Special Issue, we focus our attention on studies that describe specific and different clinical presentations of SARS-CoV-2 infection and also of post COVID-19.

Furthermore, the occurrence of severe COVID-19 is still possible in vulnerable categories of patients, such as non-responders to specific vaccines, immunocompromised patients and anti-vax people. In these cases, recent clinical improvements have been reported with use of specific antiviral drugs, such as remdesivir [3], and with new and improved ventilation support [3,4,5]. However, the most common severe complications of COVID-19 remain bacterial or fungal overinfection [6], pneumothorax and/or pneumomediastinum [7], or thrombotic complications (frequently pulmonary embolism) with or without associated molecular thrombophilia [8].

Neurological manifestations are frequent and could also appear with rare clinical signs and symptoms [9] in the subacute phase [10]. Neurological dysfunctions and cognitive impairment may be also responsible for sarcopenia and the slimming of infected patients [11].

Regarding atypical neurological symptoms and signs, cutaneous signs may appear as a rare manifestation of COVID-19 [12].

A comprehensive medical education is necessary for effective clinical practice, and day-by-day we are discovering more about the natural history of the new strain of coronavirus SARS-CoV-2 and the COVID-19 pandemic. Therefore, in this Special Issue, we present several atypical clinical aspects that may be vital for a clinical update of the natural history of this viral infection.
